# Genetic Modulation of Mercury Exposure on Perinatal and Birth Outcomes: A Systematic Review and Meta-Analysis of Gene-Environment Interactions

**DOI:** 10.3390/jox16010028

**Published:** 2026-02-06

**Authors:** Aqsa Aufa Syauqi Sadana, Saekhol Bakri, Shinji Tokonami, Eka Djatnika Nugraha, Hasnawati Amqam, Muflihatul Muniroh

**Affiliations:** 1 Master’s Program in Biomedical Science, Faculty of Medicine, Universitas Diponegoro, Semarang 50275, Indonesia; aqsa.syauqi4@gmail.com; 2 Department of Public Health Science, Faculty of Medicine, Universitas Diponegoro, Semarang 50275, Indonesia; saekholbakri@lecturer.undip.ac.id; 3 Center of Clinical Toxicology and Environmental Health (CCTEH), Faculty of Medicine, Universitas Diponegoro, Semarang 50275, Indonesia; 4 Institute of Radiation Emergency Medicine, Hirosaki University, Hirosaki-shi 036-8560, Japan; tokonami@hirosaki-u.ac.jp; 5 Research Center of Nuclear Safety, Metrology and Quality Technology, Research Organization of Nuclear Technology (ORTN), National Research and Innovation Agency (BRIN), South Tangerang 15314, Indonesia; ekad001@brin.go.id; 6 Department of Environmental Health, Faculty of Public Health, Hasanuddin University, Makassar 90245, Indonesia; hasnawati.amqam@unhas.ac.id; 7 Department of Physiology, Faculty of Medicine, Universitas Diponegoro, Semarang 50275, Indonesia

**Keywords:** genetic polymorphism, *GSTP1*, mercury, pregnancy outcomes, perinatal

## Abstract

Genetic polymorphisms can modulate susceptibility to mercury (Hg) toxicity by altering metabolic and detoxification pathways. This review evaluated the association between genetic variants, Hg exposure, and obstetric outcomes. A systematic search of Scopus, PubMed and ScienceDirect through May 2025 identified 12 eligible studies (*n* = 4995), conducted in accordance with PRISMA guidelines, with methodological quality assessed using the Newcastle–Ottawa Scale. Meta-analysis was selectively performed only for genetically and methodologically comparable studies. The most frequently examined genes were *GSTP1*, *GCLC*, *GCLM*, *GPX1*, *MT1A*, *ALAD*, and *APOE*. Meta-analysis of *GSTP1* rs1695, showed no statistically significant association between the Val105 allele and hair mercury concentrations (MD = −0.08 µg/g; 95% CI: −0.18 to 0.02; *p* = 0.13), although the direction of effect suggested a potential protective trend. Polymorphisms in other glutathione-related genes (*GCLC*, *GCLM*, and *GPX1*) were consistently associated with increased risks of small-for-gestational-age infants, preeclampsia, and impaired neurodevelopmental outcomes in offspring. In contrast, the *APOE ε4* allele appeared to be associated with reduced fetal mercury burden, whereas polymorphisms in *ALAD* and *MT1A* were linked to higher mercury levels and adverse pregnancy-related outcomes. By integrating epidemiological evidence with mechanistic insights within a gene–environment interaction framework, this review helps to address important gaps in the existing literature. These findings underscore the importance of incorporating genetic susceptibility into Hg risk assessment and precision-based prenatal interventions.

## 1. Introduction

Mercury (Hg) is a toxic heavy metal and has multiple multisystemic effects on human health [1,2]. Hg, which is frequently in the shape of methylmercury (MeHg), may accumulate in humans because they consume contaminated seafood, particularly fish, as well as through direct exposure to the environment, for example, through contact with air pollution and the skin [3,4,5]. Hg is now considered one of the top three substances of public health concern due to its effects, especially in vulnerable populations, including pregnant women and children [6]. Increased consumption of fish and marine animals by pregnant women is associated with elevated Hg levels in their hair and blood [4]. Pregnant women residing in lowland areas tend to consume fish more frequently and exhibit higher Hg levels compared to those living in highland areas [7].

Pregnancy is a time of increased susceptibility to exposure to environmental pollutants, including Hg [8]. Pregnant women exposed to Hg are at higher risk of suffering from obstetric complications, such as preeclampsia, preterm labor, and affected growth of the fetus [9,10]. The studies have reported on the effect of heavy metals such as Hg and found the influence of exposure on fetal growth, including fetal heart rate, risks of preterm birth, and low birth weight [6]. Low-dose MeHg exposure has been linked to neurotoxic effects, such as neurobehavioral parameters (fine motor function and verbal memory in children) [11], and to long-term, chronic risks in adults, including elevated chances of suffering cardiovascular and respiratory diseases [6,12].

MeHg has various cytotoxic mechanisms, animal studies have reported that MeHg increases oxidative stress by inhibiting the antioxidant capabilities of the body, increases the generation of free radicals, and leads to neurotoxicity via the expression of pro-inflammatory cytokines such as IL-6, MIP-2, and MCP-5 [13,14]. Genetic factors contribute to heavy metal exposure [15]. Genes with SNPs encoding differences in proteins are important for toxicokinetic modulation, including absorption, distribution, metabolism, and excretion of Hg, and toxicodynamic modulation, representing interaction with molecular targets and side effects [16,17,18]. Genetic polymorphisms can modulate the enzymes’ production, efficiency, and effectiveness within the MeHg metabolic pathway, which can act as the determining factor of susceptibility or resistance of the individual to Hg toxicity [19].

*GSTP1* rs1695 (Ile105Val) is a functional genetic polymorphism in the *GSTP1* gene, which encodes the phase II detoxification enzyme glutathione S-transferase pi 1 [20,21]. This polymorphism may interfere with the body’s ability to clear harmful substances such as Hg and is likely to be associated with increased susceptibility to Hg toxicity [11,22]. These polymorphisms are associated with higher or lower levels of enzyme activity and may explain differences in susceptibility to Hg exposure [23,24,25]. In addition to *GSTP1*, several other genes involved in mercury toxicokinetic and toxicodynamic have also been identified. Polymorphisms in the *GCLC*, *GCLM*, *GPX1* and *APOE* genes can affect antioxidant capacity and Hg conjugation efficiency [19], while variants in the metallothionein genes affect metal binding and tissue retention [18,26,27]. SNPs in the *MT1A* rs8052394 (Lys51Arg) gene have been related to raised Hg concentrations in tissues and the risk of biological effects, namely neurotoxic effects such as neurocognitive impairment in children and increased mild cognitive impairment (MCI) in pregnant women [26].

There are a variety of environmental and socio-economic factors that can affect Hg exposure, whereas genetics represent non-modifiable susceptibility factors [28]. To our knowledge, no studies have specifically examined a possible association between the genetic variation in the Hg detoxification pathway and pregnancy outcomes or risk of obstetric complications, nor have any shown the toxic pathway. This review aims to identify and map the synthesis of scientific evidence on the association of genetic polymorphisms with Hg detoxification pathways and obstetric outcomes in populations exposed to Hg. This review may also contribute additional information to public health policy aimed at minimizing Hg exposure in pregnant women.

## 2. Methods

This review was performed by utilizing the Preferred Reporting Items for Systematic Reviews and Meta-analyses (PRISMA) to maintain comprehensiveness and accuracy of reporting [29,30]. This review was written based on a previously published review protocol, registered at the International Prospective Register of Systematic Reviews (PROSPERO) under number CRD420251016754. During the initial literature search, the researchers used the Boolean Operators (AND, OR) to combine the keywords to maximize the search and get the most relevant literature.

### 2.1. Sources of Data and Search Strategy

A search of literature was performed in Scopus, PubMed and ScienceDirect databases from January 2015 to 31 May 2025. The search terms were conducted by using the MeSH and free-text words. The keyword combination used was as follows: (mercury exposure OR methylmercury OR mercury levels) AND (genetic polymorphism OR single nucleotide polymorphism OR SNP) AND (pregnancy outcome OR obstetric complication OR low birth weight OR preterm birth OR preeclampsia). In accordance with the PROSPERO registration, GSTP1-specific terms (GSTP1 polymorphism OR glutathione S-transferase pi 1) were additionally applied during screening and data extraction to identify studies focusing on mercury detoxification pathways. Supplementary Material Table S1 provides the detailed search strategy.

Two reviewers (AASS and SB) independently selected studies using predefined criteria. Any discrepancies in the process of selection were settled by discussion between the two reviewers until consensus was achieved. Eligible articles were included in the meta-analysis. Retrieved articles that satisfied the eligibility criteria were then screened to identify additional relevant studies through manual citation screening (snowballing) that had not been identified in the original search, which was conducted to complement the database search strategy. All citations were solicited for management with Mendeley Reference Manager, followed by the process of eliminating duplicates.

### 2.2. Eligibility Criteria

The eligibility criteria were observational (cohort, case-control, or cross-sectional) studies that analyzed the association between Hg exposure under biomarker blood, hair, urine, or cord tissue and genetic polymorphisms. We included studies published in English from January 2015 to March 2025, and excluded articles published before 2015. In addition, studies that applied both acceptable techniques for measuring the Hg levels (for example, PCA-RFLP, qPCR, or DNA sequencing) and genotyping, as well as reported the frequencies of genotypes, were included in the review.

Studies that did not measure body burdens of Hg in individuals or did not report genotype information were excluded. Also, animal studies, in vitro studies, and experimental models were excluded. Review, meta-analysis, case report, editorial, conference abstract, and studies with unextractable or insufficient data were also excluded.

### 2.3. Study Process

**Figure 1:**
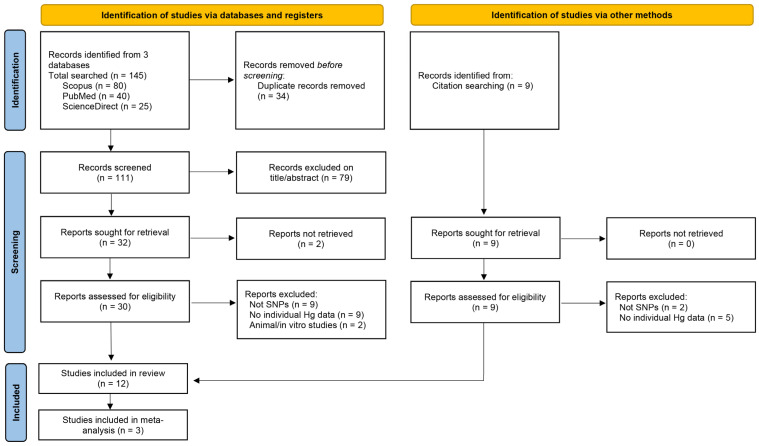
PRISMA Flow Diagram. Flow diagram of the study identification and selection process. PRISMA 2020 checklists are provided in the Supplementary Materials Tables S3 and S4.

After finishing the initial search, three reviewers (AASS, SB, and EDN) screened the studies through titles and abstracts for those meeting the inclusion criteria. Full texts of the articles that passed the first stage were searched. Any discordant views during the screening process were resolved through discussion or the opinion of MM and ST. The PRISMA flowchart of studies, including the number of identified records, screened records, eligible for full-text review, excluded at the full-text level, and included in the meta-analysis, is depicted in Figure 1.

A total of 145 articles were found, as shown in Figure 1. There were 34 duplicative studies and 9 additional records identified through citation searches. During the screening phase, 111 records from the databases were screened by title and abstract, resulting in the exclusion of 79 studies. Thirty-two reports were sought for retrieval, of which two could not be obtained, leaving 30 reports assessed for eligibility. Most research was disqualified for not addressing the genetics of participants. Finally, studies that did not report data on SNPs or Hg exposure were excluded. Twelve studies were considered eligible for the inclusion review, and three were included in meta-analyses. Detailed database search results are available as Supplementary Files S1–S3.

### 2.4. Extraction and Quality Assessment

After the final selection, the full-text articles were retrieved, and the desired data was collected. The recorded data included first author names, years, countries, population, methods, and key findings. The data also included population characteristics, such as the number of populations, sex ratio, average age of subjects, body mass index (BMI) and Hg exposure sources. Furthermore, gene types, SNPs, obstetric outcomes, and Hg levels were also recorded. For each study (where the mean was obtained from the geometric mean), data were extracted or transformed to mean difference (MD) with standard deviation (SD), and the transformation was mathematically applied based on statistical techniques [31].

The methodological quality of the included studies was measured by the Newcastle-Ottawa Scale (NOS) for observational studies [32]. Study quality is assessed using the NOS, focusing on three key points of selection, comparability, and result. Studies with a score ≥ 7 were regarded as high-quality, and studies with a score < 7 were all considered as high-risk of bias and were removed from the analysis of sensitivity.

### 2.5. Meta-Analysis

RevMan 5.4 was used to perform a meta-analysis of Hg levels between genotype comparison by MD and 95% CI [30]. If there was no significant heterogeneity (I^2^ < 50%), the fixed-effects model was chosen, and when there was high heterogeneity (I^2^ ≥ 50%), the random-effects model was chosen. Between-study heterogeneity was tested by the I^2^ statistic and the Cochran’s Q test, with I^2^ values < 25% and 25–50% representing low and moderate, and > 50% representing high between-study heterogeneity. Publication bias was determined by performing the funnel plot and Egger’s test (*p* < 0.05 suggested potential publication bias).

## 3. Results

### 3.1. Identification and General Characteristics of the Selected Study

Based on the search results in 3 databases, 12 articles were included based on the selection criteria and the contained data with *n* = 4955 participants who were predominantly from Brazil (4 studies, including Brazilian Amazon, Brazil, South America), Italy (2 studies), and other countries, Thailand, USA, Seychelles, China, Jamaica, Austria, Slovakia, and Africa, one study each. The populations were pregnant women, children, rural and riverine communities, and urban workers. The most utilized study design was cross-sectional (seven studies), followed by cohort (five studies), and 1 case-control study. Hg exposure is predominantly via consumption of fish and, in part, through dental amalgam or occupational exposure (Table 1).

The NOS was used to evaluate the quality and eligibility of the included studies. Methodological quality, which scored between 7–9 out of a possible maximum of 9 stars, was similar. General strengths included well-defined exposures, outcome assessments, and cohort representation. These benefits include an easy identification of Hg levels. Nevertheless, several studies were unclear in adjusting for potential confounding and describing the amount of follow-up. The NOS scores for each of the studies are shown in Supplementary Material Table S2.

### 3.2. Genetic Polymorphisms Associated with Hg Levels and Obstetric Outcomes

Biomarkers used to measure Hg levels are blood, hair, urine, and umbilical cord. The threshold level is different for each biomarker [42,43]. In rural villages, Hg levels have been found to go well beyond the limit. Pareni et al. (2021) found high Hg levels in the Brazilian population above the hair biomarker cut-off for the WHO (>2 µg/g) [37]. On the other hand, populations living in cities usually have low levels of Hg [4,44], and a few individuals are at risk when they present some sensitive allele or genotype [26,39]. The Hg contents and SNP data of genes in each study are presented in Table 2.

The genes summarized in Table 3 represent key components of the mercury toxicokinetic and toxicodynamic pathways. Glutathione-related genes are involved in antioxidant defense and phase II detoxification, while metallothionein genes function as metal-binding proteins that regulate intracellular mercury sequestration. *ALAD* participates in heme biosynthesis and heavy metal binding, while *APOE* is involved in lipid transport and placental mercury transfer.

The correlations of single-nucleotide polymorphisms (SNP) and obstetric complications are summarized in Table 3. Eighteen SNPs in 14 genes, identified in the present study, were correlated with Hg. The studies were classified depending on the pathway mediated by their activity, with those including the glutathione pathway, the metallothionein (MT) pathway, and the activity of transporters and other metabolic enzymes. SNPs in GSH and MT pathways presented significant associations with Hg levels in blood, hair, and urine [21,26,35] Three SNPs (*GSTP1* rs1695 (Ile105Val), *MT1A* rs8052394 (Lys51Arg), and *MT1M* rs2270836) were repeatedly related to high Hg in all studies [5,26]. In pregnant women, the presence of the *APOE* ε4+ allele is associated with reduced fetal Hg accumulation, suggesting its role as a protective allele [38]. In addition, gene-obstetric complication interactions were also identified in the *ALAD* rs1800435, and pregnant women carrying this gene were correlated with placental transfer efficiency and susceptibility to SGA [38]. The SNPs in GSH and MT detoxification genes were significantly related to higher Hg accumulation and adverse fetal outcomes (lower child cognition), while it depended on the specific allele or genotype [22].

### 3.3. Associate of GSTP1 rs1695 Polymorphisms and Hair Hg Levels

Three studies reported the same results regarding the *GSTP1* rs1695 (Ile105Val) gene and Hg levels in hair [11,33,36]. The studies were also selected for a second meta-analysis to investigate the relationship of the *GSTP1* rs1695 polymorphism with hair Hg levels. All three studies compared individuals with the *GSTP1* rs1695 Ile105/Ile105 (AA) genotype to Val105 allele carriers (Ile105/Val105 and Val105/Val105; AG/GG), with air as well as hair as a biomarker for MeHg exposure and thus capture the response on a similar biological background. The number per genotype in the Parajuli et al. (2016) [33] study was calculated assuming HW and a Minor Allele Frequency (MAF) of 0.33. This estimation was predicted because the real data were not provided by the authors. The random-effects meta-analysis yielded a non-significant MD (MD = −0.08 µg/g; 95% CI: −0.18 to 0.02; *p* = 0.13).

**Figure 2:**
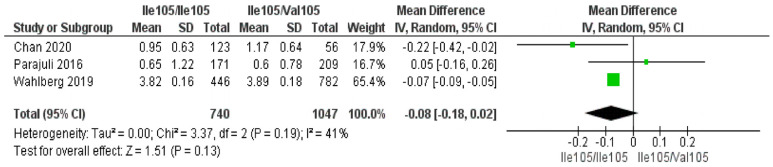
Forest plot of *GSTP1* rs1695 polymorphism and hair Hg levels (µg/g), comparing *GSTP1* Ile105/Ile105 and Ile105/Val105 genotypes. Mean differences from individual studies and the pooled estimate (diamond centerline), with corresponding 95% confidence intervals, are depicted. References: Chan (2020) [11], Parajuli (2016) [33], Wahlberg (2019) [36].

The results were not statistically significant but indicated an inverse trend for hair Hg levels and the AA (Ile105) genotype compared to individuals carrying the G (Val105) allele. There was some heterogeneity among the studies (I^2^ = 41%, *p* = 0.19). The meta-analysis results of the *GSTP1* rs1695 polymorphism and hair Hg levels (µg/g) are presented in Figure 2.

## 4. Discussion

Mercury is a well-documented environmental toxin that has neurotoxic, immunotoxic, and reproductive effects [45,46,47]. Its distribution in the biological system depends on its chemical form (elemental, inorganic, or methylmercury) and on the exposure route, such as dietary intake (mainly fish consumption), occupational sources, and dental amalgam [48,49]. Hg exposure in humans due to the gastrointestinal route occurs during eating seafood [7]. Recent research, however, underscores the importance of genetic factors, particularly polymorphisms in genes involved in Hg transport, detoxification, and cellular response, in accounting for the significant interindividual variability observed in Hg metabolism and toxicity [50,51,52].

This systematic review and meta-analysis emphasize that polymorphisms contribute to Hg uptake and the toxicity effects, especially during pregnancy and birth. The studies that were part of this systematic review were conducted in different geographical areas, including Brazil, Italy, Vietnam, China, Jamaica, Austria, and the USA, and had various populations. This review concentrates on genetic polymorphisms related to the Hg detoxification pathways, especially the glutathione (GSH) and metallothionein (MT) pathways [11,35] Genes including *GCLC* (rs17883901), *GCLM* (rs41303970), and *GSTP1* (rs1695) have been implicated in modifying blood or hair Hg concentrations [36]. The *GCLM* gene TT allele is linked to less Hg than CC or CT, suggesting enhanced detoxification capability [36].

The *GSTP1* rs1695 polymorphism reveals intriguing yet conflicting insights. According to Silva et al., there is a correlation between blood Hg levels and a reduction in neurological symptoms [5]. In contrast, Wahlberg et al. (2018) identified a link between Hg levels and decreased mental development in children [36]. These findings highlight a complex relationship between detoxification capacity and developmental disorders. The *GSTP1* rs1695 (Ile105Val) gene serves as a representative example of a phase II detoxification enzyme, which plays a crucial role in the conjugation of glutathione to MeHg [53,54,55]. The G (Val105) allele has been associated with decreased Hg levels in previous studies, a finding that this meta-analysis supports [53]. While the effect has not yet achieved statistical significance, its direction remains biologically plausible. The enzyme activity altered by this polymorphism plays a role in mediating the extent of Hg elimination via bile and urine [56,57,58].

**Figure 3:**
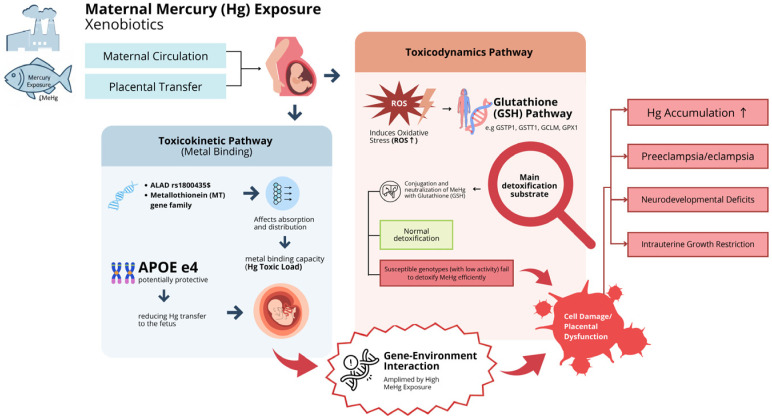
Genetic modulation pathways and Hg toxicity on perinatal outcome interactions.

Meta-analysis indicated that there was no significant association between the G allele of *GSTP1* rs1695 polymorphism and lower Hg levels in hair [11,33,36]. The results indicate that *GSTP1* is responsible for MeHg conjugation via the glutathione pathway before excretion [22,59,60,61]. This lack of significance may be confounded by variation in fish consumption, or exposure to other heavy metals, the nutritional status represented by selenium intake, and differences in MAF among populations. The moderate heterogeneity (I^2^ = 41%) found is also a sign of uncontrolled confounding. Moreover, differences in the exposure to other heavy metals (lead and cadmium) or nutritional status (selenium and omega-3 fatty acids) can be additional confounders that are not always properly considered in the primary studies [33]. The comprehensive mechanism of genetic modulation of Hg toxicity causing perinatal complications is summarized in the flow diagram in Figure 3.

Figure 3 illustrates the mechanisms by which genetic polymorphisms influence individual susceptibility to MeHg during pregnancy. Toxicity is determined by the interaction between toxicokinetic and toxicodynamic pathways. Other glutathione-related pathways and included in the toxicodynamic pathway, *GCLC* and *GCLM* were also identified as potential modulators of Hg levels [36]. These genes encode the catalytic subunit of the most critical enzyme in controlling tissue levels of GSH as an antioxidant and detoxification substrate, glutamate-cysteine ligase [62,63]. Specific genetic polymorphisms such as *GCLM* rs41303970 contribute to the reduction of fetal GSH levels [36], increased oxidative stress enhancement, and the magnification of Hg accumulation. This effect is particularly significant during pregnancy due to the increased demand for antioxidants, which impacts placental function and may elevate the risk of obstetric complications such as preeclampsia and intrauterine growth restriction (IUGR) [64]. The rs1050450 of the *GPX1* gene involved in reducing ROS by converting H_2_O_2_ to H_2_O, is important [20]. The mutations of *GPX1* can, to some extent, decrease the antioxidant defense ability of the organism and aggravate oxidative damage by Hg, which may lead to placental dysfunction, endothelial damage, and inhibited fetal growth [65,66].

Apart from the GSH pathway, metallothionein families were also known to be important for heavy metal ion binding and detoxification, which were mostly encoded by genes like *MT1A*, *MT1M*, and *MT2A* [26,27]. In addition, MT genes, such as *MT1M* (rs2270836) and *MT1A* (rs8052394), in the metallothionein family also affect Hg levels and neurological symptoms [26]. MTs combine with heavy metal ions, decrease their bioavailability, and are vitally involved in low-dose chronic exposure [10,25]. This is relevant, especially in human low-dose chronic exposures, as in people living in coastal communities or among fish consumers in inland areas [67,68]. MT has also been shown to be involved in the antioxidant defense system, as well as playing a significant role in protecting placental cells against injury caused by heavy metals, which is important for pregnancy [69].

Additionally, metallothionein (MT) serves as a repository for heavy metals such as Hg^2+^ and Cd^2^, and it is instrumental in maintaining Zn/Cu homeostasis, a function that is particularly vital during pregnancy [70]. The *MT1A* rs8052394 polymorphism (Lys51Arg), particularly the *MT1A* 51Arg variant, is associated with decreased metal-binding capacity, which then results in increased Hg load and systemic oxidative stress [26]. The variant rs8052394 at *MT1A* was related to elevated Hg and higher neurological symptoms [26]. It is linked to fetal neurodevelopmental disability and the risk of mothers’ mild cognitive impairment (MCI) and children’s neurocognitive impairment [71].

The *ALAD* rs1800435 polymorphism (Lys59Asn), encoding the enzyme, which is a critical enzyme in heme biosynthesis (catalyzing the conversion of ALA to porphobilinogen), is also associated with the removal of heavy metals [38]. The *ALAD* isoenzyme exhibits a pronounced affinity for Hg, particularly among individuals carrying the Asn59 variant of *ALAD* (*ALAD*-2) [32]. This interaction leads to the inhibition of heme synthesis, reduced oxygen delivery, and an increased metal burden. During pregnancy, these impairments adversely affect placental efficiency in oxygen transfer and are associated with an elevated risk of small for gestational age (SGA) infants and fetal neurotoxicity [72,73] The *ALAD* gene (rs1800435), previously studied mostly in native populations, demonstrated a raised blood ratio of Hg and a greater risk of neurotoxicity in carriers of the *ALAD* Asn59 variant [38].

On the other hand, the *APOE* ε4 allele in pregnant women was found to influence Hg retention and its potential risk for the fetus, thus possibly protecting mothers from Hg transfer through the placenta [38,74]. Palir et al. (2023) demonstrated that ε4 carriers presented reduced fetal Hg levels, possibly because of the efficiency of lipid transfer and the protective effect against oxidative stress [38]. This discovery suggests a potential systemic biomarker of fetal susceptibility to the impact of Hg [16,19].

Other xenobiotic metabolism and transplacental transfer-related genes, including *ABCG2*, *UGT2B15*, and CYP3A family (*CYP3A4*, *CYP3A5*, *CYP3A7*), can be interesting [75]. CYP3A gene SNPs can influence Hg and even children’s MDI scores via putative interplay in fish nutrient metabolism and heavy metal detoxification [39]. An efflux transporter, *ABCG2* rs2231142, can influence Hg excretion from placental cells, whereas *UGT2B15* rs1902023, by conjugating the toxic compounds introduced in the placenta, modifies their effect on the fetus [76].

Overall, the research results demonstrate the importance of not only the exposure level, but also the individual genetic predisposition to Hg, in relation to metabolic pathway, oxidative stress, and excretion efficiency [77,78]. In pregnant women and other vulnerable groups, these effects compound to increase the risk of pregnancy-related complications and long-term adverse pregnancy outcomes such as fetal growth restriction, preeclampsia, and neurodevelopmental deficits in offspring [47,79]. Therefore, genetic and molecular strategies are very much needed for early screening of high-risk populations and personalized public health strategies, including genotype-guided screening and individualized recommendations for fish consumption.

The biological functions uncovered also support the roles of the systemic axis, including heme biosynthesis, GSH metabolism, metal binding, and xenobiotic excretion [22,46]. The interactions of genes with toxic substances, such as Hg, are complex and context-specific, and they are addressed using a transdisciplinary approach cutting across the disciplines of molecular toxicology, fetal medicine, nutrigenomics, and environmental health policy. During pregnancy, many investigations, including those of Gundacker et al. (2021) and Palir et al. (2023), have reported an influence of maternal genotype on the transfer of Hg to the fetus and obstetric outcomes, such as SGA birth [38,41]. Wahlberg et al. (2018) demonstrated that variations in the GSH pathway of the mothers can modify the kinetics of Hg, as well as influence the neurocognitive development of the child [36]. These findings highlight the need to consider gene-environment interactions when evaluating obstetrical complications and their long-term effects for children.

The present review is the first to demonstrate the relationship of genetic polymorphisms and Hg exposure in various populations and biomatrices (blood, hair, urine, and umbilical cord). This comprehensive framework can facilitate the initial step for characterizing at-risk populations and establishing genomics-based preventive interventions. Yet there are a few shortcomings in this study. Heterogeneity of biomarker categories, units of measure, and SNP reporting has impeded our ability to generalize findings. Most studies used were cross-sectional in design, and a small sample size precluded their potential to infer a causal relationship. As such, prospective large longitudinal cohort studies with standardized methods of biomarker measurement and consistent genotyping are needed.

Beyond genetic susceptibility, environmental exposure itself remains an important determinant of health risks. Mercury has long been known for its adverse health effects, particularly among vulnerable populations such as pregnant women, necessitating comprehensive and collaborative strategies to address environmental contamination. Dietary exposure through seafood consumption represents a major pathway of chronic heavy metal exposure during pregnancy [80], especially in coastal populations. Currently, environmental contamination is not limited to mercury. Other environmental pollutants, particularly in mining areas, have been reported to pose long-term public health risks [81,82]. In Indonesia, chronic low-dose environmental exposure may contribute to adverse health outcomes through mechanisms such as oxidative stress [83,84], potentially exacerbating risks during pregnancy. This context underscores the importance of an integrative approach in evaluating environmental exposures and their impact on maternal health.

An important finding from this review is that GSTP1 rs1695 consistently showed a trend toward a protective association against Hg retention. This trend, although not statistically significant, has clinical and toxicological importance. In-depth studies in relation to dominant vs. recessive genetic models, adjusting for exposure via fish consumption, and functional outcome quantifications (neurocognitive injury, complications in pregnancy) in the context of gene-environment interactions are warranted in the future. Integrating these analyses can significantly assist precision public health, especially with respect to pregnant women and children, who are the most susceptible to Hg exposure.

## 5. Conclusions

In conclusion, genetic variations within the glutathione detoxification pathway, heme biosynthesis, metal binding, and xenobiotic metabolism, specifically involving the *GSTP1*, *GCLM*, *MT1A*, *ALAD*, and *APOE* genes, significantly influence Hg accumulation and its effects on obstetric health. Certain polymorphisms may modulate detoxification efficiency, antioxidant capacity, and transplacental transfer efficiency, thereby elevating the risk of pregnancy complications such as preeclampsia, small for gestational age (SGA), intrauterine growth restriction (IUGR), and neurocognitive developmental issues in offspring. Although not all effects reached statistical significance, the consistent direction of these effects underscores the importance of genetic factors in Hg susceptibility. Consequently, it is imperative to incorporate genetic information into prenatal screening and environmental health models to mitigate the long-term effects of heavy metal exposure, particularly in high-risk subpopulations, such as pregnant women and their fetuses.

## Figures and Tables

**Table 1:** General characteristics of the included studies.

No	Countries	Population	n	Sex	Age	BMI (kg/m^2^)	Methods	Key Findings	Refs.
1	Brazil	Adult Rural	107	47.6% Male	23.2 ± 6.13(12–35)	23.7 ± 3.08	Cross Sectional	Carriers of the *GSTP1* rs1695 Val105 (G) allele showed lower Hg levels and milder neurological symptoms.	[5]
2	USA	Urban adults	380	62.6% Male	54.8 ± 11.4	-	Cross sectional	*GSTP1* rs1695 Val105 allele carriers have lower blood Hg concentrations.	[33]
3	Brazilian Amazon	Rural Children	82	40% Male	0–11 ^a^	35 (89.7) ^b^	Cross sectional	The *GSTP1* rs1695 Val105 allele was associated with lower Hg levels and a reduced risk of neurological symptoms	[34]
4	Jamaica	Urban children	266	81.6% Male	2–8 ^c^	-	Case-control	Individuals with the *GSTP1 rs1695* Ile105/Ile105 genotype showed higher Hg concentrations (0.73 µg/L) compared with Val allele carriers.	[35]
5	Africa	Maternal	1536	Female	Not stated	-	Cohort Study	Maternal GSH-pathway genes influence MeHg metabolism and may modulate developmental outcomes	[36]
6	Brazil	Rural Children	101	39.6% Male	6.4 ± 4.4 (0–14)	18.8 ± 1.9 ^d^; 16.0 ± 1.3 ^e^	Cross sectional	Children with *ALAD* rs1800435 CG genotype have high Hg levels and chronic neurological symptoms.	[37]
7	Italy	Pregnant Women	873	Female	32.7 ± 4.6 (18–44)	22.5 ± 0.1	Cohort Study	*APOE* ε4 allele protects the fetus from Hg accumulation.	[38]
	Italy	Newborn	619	52.3% Male	39.5 ± 1.4 weeks	-	-		
8	Seychelles	Urban children	211	48.8% Male	2.3 ± 1.2	-	Cohort Study	*CYP3A* family *(CYP3A4*, *CYP3A5*, *CYP3A7)* variation affects cord Hg-induced neurodevelopment.	[39]
9	Thailand	Urban adults	106	65.1% Male	58.8 ± 3.0	24.8 ± 3.7	Cross sectional	*MT1A* rs8052394 (Lys51Arg) is associated with MCI and high Hg level	[26]
10	China	Urban children	179	54.3% Male	4.6 (4–5.3)	14.9 (14–16)	Cohort Study	Minor alleles *GCLC* rs17883901, *GPX1* rs1050450 (Pro198Leu), and *MT1M* rs9936741 were associated with lower Hg levels.	[11]
11	Brazil (South America)	Riverine-Adults	395	Not Stated	40.5 (18–87)	24	Cross sectional	Polymorphisms in detoxification/antioxidant genes modulate Hg/Pb burden and oxidative stress	[40]
12	Austria (Vienna)	Pregnant women ^f^	100	Female	31 (18–43)	28 (20–37)	Cohort Study	Gene variants influence placental transfer efficiency and the risk of SGA birth.	[41]
	Slovakia	Pregnant women ^g^	100	Female	31 (18–43)	27 (20–39)			

^a^ 48% of study participants were 0–4 years old; ^b^ BMI eutrophy with Z-score obtained for BMI for age measure in children older than 5 years; ^c^ 30% of study participants were older than 3 years; ^d^ Including children older than 10 years; ^e^ Referring to the 6–10 years age group; ^f^ 100 mother-child pairs in Bratislava; ^g^ 100 mother-child pairs in Vienna.

**Table 2:** Genetic polymorphisms and Hg levels.

No	Genes	dbSNP ID	Hg Level				Hg Category ^a^	Refs.
			Umbilical Cord (µg/L)	Blood (µg/L)	Hair (µg/g)	Urine (µg/L)		
1	*ABCG2*	rs2231142	0.41 (0.31–0.45)	-	-	-	Low	[41]
2	*ALAD*	rs1800435	-	-	10.9 ± 5.6	-	High	[37]
			-	39.8	–	–	High	[40]
			0.41 (0.31–0.45)	-	-	-	Low	[41]
3	*APOE*	rs7412	-	1.95 (1.62–2.34)	-	-	Low	[38]
		rs429358	3.35 (2.83–3.96)	-	-	-	Low	
4	*CYP3A4*	rs2740574	39.3 ± 25	-	5.8	-	High	[39]
5	*CYP3A5*	rs776746	-	-	8.3	-	High	
6	*CYP3A7*	rs2257401	-	-	5.43	-	High	
7	*GCLC*	rs17883901	-	3.7 ± 3.9	0.6 ± 1.0	1.3 ± 1.8	Medium	[33]
		rs17883901	-	-	0.97 (0.62–1.51)	-	Medium	[11]
		rs761142	34.44 (32.43–36.45)	18.43 (17.51–19.35)	4.12 (3.83–4.42)	-	High	[36]
8	*GCLM*	rs41303970	34.84 (33.13–36.55)	18.28 (17.50–19.06)	4.07 (3.82–4.32)	-	High	[36]
9	*GPX1*	rs1050450	-	-	1.02 (0.66–1.51)	-	Medium	[11]
10	*GSTP1*	rs1695	-	-	≤6.0 (34.4%) ^b^	-	-	[5]
			-	3.6 ± 3.7	0.6 ± 0.9	1.3 ± 1.6	Medium	[33]
			-	-	≤5.5 (40%) ^c^	-	-	[34]
			-	1.0 (0.7–1.5)	-	-	Low	[35]
			33.75 (31.54–35.95)	18.44 (17.44–19.44)	3.82 (3.50–4.14)	-	High	[36]
		rs1138272	0.56 (0.05–5.68)	-	-	-	Low	[41]
11	*GSTT1*	Deletion	1.33 (0.52–3.78)	-	-	-	High	[41]
12	*MT1A*	rs8052394	-	6.3 (0.8–27.6)	-	-	High	[26]
13	*MT1M*	rs2270836	-	-	1.02 (0.70–1.52)	-	Medium	[11]
14	*MT2A*	rs10636			1.00 (0.65–1.50)	-	Moderate	[11]
15	*UGT2B15*	rs1902023	1.25 (0.63–4.05)	-	-	-	High	[41]

^a^ Categories of Hg levels based on established safe limits based on each biological matrix: blood ≥ 5.8 µg/L is categorized as high based on US EPA, hair >2 µg/g is categorized as high based on WHO, and urine >4 µg/L is categorized as high based on WHO guidelines; ^b^ The researchers grouped Hg levels ≤ 6.0 µg/g vs. ≥6.0 µg/g; ^c^ The researchers grouped Hg levels < 5.5 µg/g vs. ≥5.5 µg/g.

**Table 3:** Hg detoxification gene pathway linked to obstetric and birth outcomes.

No	Genes	dbSNP ID	Gene Activity Pathway	Outcome of Obstetric/Birth	Refs.
1	*ABCG2*	rs2231142	Xenobiotic transporter (efflux)	Not Reported	[41]
2	*ALAD*	rs1800435, rs1800436	Heme biosynthesis, heavy metal detoxification	Increased risk of Small for Gestational Age	[37,40]
3	*APOE*	rs7412, rs429360	Lipid metabolism & neuroprotection	Potential fetal protection from Hg if the mother carries the ε4 allele	[38]
4	*CYP3A4*	rs2740574	Cytochrome P450 (xenobiotic metabolism)	Effects on children’s neurocognitive response	[39]
5	*CYP3A5*	rs776746	Cytochrome P450	Fixed association with Hg neurotoxicity	[39]
6	*CYP3A7*	rs2257401	Cytochrome P450	Fixed association with Hg neurotoxicity	[39]
7	*GCLC*	rs761142, rs17883901	Glutathione (GSH) synthesis	Not reported	[11,36]
8	*GCLM*	rs41303970	Glutathione (GSH) synthesis	Affects Hg levels and oxidative stress	[11,36]
9	*GPX1*	rs1050450	GSH-peroxidase (antioxidant)	Not Reported	[11]
10	*GSTP1*	rs1695	GSH transferase (phase II detoxification)	Decreased children’s MDI score	[5,36]
11	*MT1A*	rs8052394	Metallothionein (heavy metal binder)	Modified MCI & increased Hg tertile	[26]
12	*MT1M*	rs9936741, rs2270836	Metallothionein	Not Reported	[11]
13	*MT2A*	rs10636	Metallothionein	Not Reported	[11]
14	*UGT2B15*	rs1902023	Glucuronidation (phase II metabolism)	Effects on placental transfer and risk of SGA infants	[41]

MDI: mental development index, MCI: mild cognitive impairment, SGA: small for gestational age.

## Data Availability

No new data were created or analyzed in this study. Further inquiries can be directed to the corresponding authors.

## Supplementary Materials

The following supporting information can be downloaded at: https://www.mdpi.com/article/10.3390/jox16010028/s1, Table S1. Literature search strategy; Table S2. The NOS scores for each of studies; Table S3. PRISMA 2020 for abstracts checklist; Table S4. PRISMA 2020 checklist; Detailed database search results are available as Supplementary Files S1–S3.

## Abbreviations

*ALAD*	δ-aminolevulinic acid dehydratase
*APOE*	Apolipoprotein E
ASD	Autism Spectrum Disorder
Cd^2+^	Cadmium ion
CI	Confidence Interval
Cu	Copper
DNA	Deoxyribonucleic Acid
*GCLC*	Glutamate-Cysteine Ligase Catalytic Subunit
*GCLM*	Glutamate-Cysteine Ligase Modifier Subunit
*GPX1*	Glutathione Peroxidase 1
GSH	Glutathione
*GSTP1*	Glutathione S-Transferase Pi 1
Hg	Mercury
Hg^2+^	Mercury ion
HW	Hardy-Weinberg equilibrium
MAF	Minor Allele Frequency
MCI	Mild Cognitive Impairment
MD	Mean Difference
MDI	Mental Development Index
MeHg	Methylmercury
*MT1A*	Metallothionein 1A genes
*MT1M*	Metallothionein 1M genes
*MT2A*	Metallothionein 2A genes
NOS	Newcastle–Ottawa Scale
PCR	Polymerase Chain Reaction
PRISMA	Preferred Reporting Items for Systematic Reviews and Meta-Analyses
RFLP	Restriction Fragment Length Polymorphism
qPCR	Quantitative Polymerase Chain Reaction
SD	Standard Deviation
SGA	Small for Gestational Age
SNP	Single Nucleotide Polymorphism
Zn	Zinc
