# Supplementary material for: Genetic Modulation of Mercury Exposure on Perinatal and Birth Outcomes: A Systematic Review and Meta-Analysis of Gene-Environment Interactions
Source: J Xenobiot. 2026 Feb 6;16(1):28. doi: 10.3390/jox16010028 (PMC12921806; doi:10.3390/jox16010028)
Supplement: Supplementary file 1 [file jox-16-00028-s001.zip › jox-4098527-supplementary.pdf]

Supplementary Material

Table S1. Literature search strategy

Database	Search strategy
PubMed	(("Mercury"[Mesh] OR mercury exposure OR methylmercury OR mercury levels)) AND ("genetic polymorphism" OR "single nucleotide polymorphism" OR SNP) AND ("pregnancy outcome" OR "obstetric complication" OR "low birth weight" OR "preterm birth" OR preeclampsia)
Scopus	TITLE-ABS-KEY (mercury exposure OR methylmercury OR mercury levels) AND TITLE-ABS-KEY (genetic polymorphism OR single nucleotide polymorphism OR SNP) AND TITLE-ABS-KEY (pregnancy outcome OR obstetric complication OR low birth weight OR preterm birth OR preeclampsia)
ScienceDirect	("mercury exposure" OR methylmercury OR "mercury levels") AND ("genetic polymorphism" OR "single nucleotide polymorphism" OR SNP) AND ("pregnancy outcome" OR "obstetric complication" OR "low birth weight" OR "preterm birth" OR preeclampsia)

Note: GSTP1-specific terms (“GSTP1 polymorphism” OR “glutathione S-transferase P1”) were applied during screening and data extraction in accordance with the PROSPERO registration.

Table S2. The NOS scores for each of the studies

Study	Selection				(5) Comparability		Outcome			Total Score
	(1) Rep. Exposed Cohort	(2) Selection non-exposed	(3) Ascertainment of exposure	(4) Outcome Not Present at Start	Main factor	Additional factor	(6) Outcome Assessment	(7) Follow-Up Long Enough	(8) Adequacy of Follow-Up	
[32]	★	★	★	★	★	0	★	★	0	7/9
[33]	★	★	★	0	★	0	★	★	★	7/9
[5]	★	★	★	0	★	0	★	★	★	7
[34]	★	★	★	★	★	★	★	★	★	9/9
[35]	★	★	★	★	★	★	★	★	0	8/9
[36]	★	★	★	★	★	0	★	★	★	8/9
[25]	★	★	★	★	★	0	★	★	★	8/9
[10]	★	★	★	★	★	★	★	★	★	9/9
[37]	★	★	★	0	★	★	★	★	★	8/9
[38]	★	★	★	★	★	0	★	★	★	8/9
[39]	★	★	★	★	★	★	★	★	0	8/9
[40]	★	★	★	0	★	0	★	★	★	7/9

Table S3. PRISMA 2020 for Abstracts Checklist

Section and Topic	Item #	Checklist item	Reported (Yes/No)
**TITLE**			
Title	1	Identify the report as a systematic review.	YES
**BACKGROUND**			
Objectives	2	Provide an explicit statement of the main objective(s) or question(s) the review addresses.	YES
**METHODS**			
Eligibility criteria	3	Specify the inclusion and exclusion criteria for the review.	NO
Information sources	4	Specify the information sources (e.g. databases, registers) used to identify studies and the date when each was last searched.	YES
Risk of bias	5	Specify the methods used to assess risk of bias in the included studies.	YES
Synthesis of results	6	Specify the methods used to present and synthesise results.	YES
**RESULTS**			
Included studies	7	Give the total number of included studies and participants and summarise relevant characteristics of studies.	YES
Synthesis of results	8	Present results for main outcomes, preferably indicating the number of included studies and participants for each. If meta-analysis was done, report the summary estimate and confidence/credible interval. If comparing groups, indicate the direction of the effect (i.e. which group is favoured).	YES
**DISCUSSION**			
Limitations of evidence	9	Provide a brief summary of the limitations of the evidence included in the review (e.g. study risk of bias, inconsistency and imprecision).	NO
Interpretation	10	Provide a general interpretation of the results and important implications.	YES
**OTHER**			
Funding	11	Specify the primary source of funding for the review.	NO
Registration	12	Provide the register name and registration number.	NO

From: Page MJ, McKenzie JE, Bossuyt PM, Boutron I, Hoffmann TC, Mulrow CD, et al. The PRISMA 2020 statement: an updated guideline for reporting systematic reviews. BMJ 2021;372:n71. doi: 10.1136/bmj.n71. This work is licensed under CC BY 4.0. To view a copy of this license, visit https://creativecommons.org/licenses/by/4.0/

Table S4. PRISMA 2020 Checklist

Section and Topic	Item #	Checklist item	Location where item is reported
**TITLE**			
Title	1	Identify the report as a systematic review.	Title, Page 1
**ABSTRACT**			
Abstract	2	See the PRISMA 2020 for Abstracts checklist.	The abstract was prepared in accordance with the PRISMA 2020 for Abstracts checklist, including clear reporting of objectives, information sources, risk of bias assessment, synthesis methods, main results, and key implications.<br>Abstract, Page 1, Line 26-43
**INTRODUCTION**			
Rationale	3	Describe the rationale for the review in the context of existing knowledge.	The Introduction, Paragraphs 2-3, discusses the genetic and environmental susceptibility of perinatal conditions to mercury. This includes highlighting the lack of integrated analysis of perinatal gene-environment interactions in previous research.
Objectives	4	Provide an explicit statement of the objective(s) or question(s) the review addresses.	Introduction, Paragraph 4, Lines 85-92
**METHODS**			
Eligibility criteria	5	Specify the inclusion and exclusion criteria for the review and how studies were grouped for the syntheses.	Methods section, Point 2.2. Eligibility Criteria, Page 3, Lines 119-131
Information sources	6	Specify all databases, registers, websites, organisations, reference lists and other sources searched or consulted to identify studies. Specify the date when each source was last searched or consulted.	In the Sources of data and search strategy section, Lines 105-118 have explained the information sources.

Section and Topic	Item #	Checklist item	Location where item is reported
			PROSPERO Number CRD420251016754.
Search strategy	7	Present the full search strategies for all databases, registers and websites, including any filters and limits used.	The full electronic search strategies, including keywords, Boolean operators, databases searched (PubMed, Scopus, Web of Science, and ScienceDirect), time limits (January 2015–March 2025), and language restrictions, are reported in the Methods section (Section 2.1).
Selection process	8	Specify the methods used to decide whether a study met the inclusion criteria of the review, including how many reviewers screened each record and each report retrieved, whether they worked independently, and if applicable, details of automation tools used in the process.	Study selection was conducted independently by two reviewers at the title/abstract stage (Line 112-116) and by three reviewers at the full-text stage. Disagreements were resolved through discussion and consensus, as described in Section 2.3.
Data collection process	9	Specify the methods used to collect data from reports, including how many reviewers collected data from each report, whether they worked independently, any processes for obtaining or confirming data from study investigators, and if applicable, details of automation tools used in the process.	Data extraction was independently performed by multiple reviewers using a predefined data extraction form, with discrepancies resolved by discussion, as detailed in Section 2.4, Page 4
Data items	10a	List and define all outcomes for which data were sought. Specify whether all results that were compatible with each outcome domain in each study were sought (e.g. for all measures, time points, analyses), and if not, the methods used to decide which results to collect.	The primary outcomes included mercury biomarker levels (blood, hair, urine, cord tissue) and obstetric outcomes such as preeclampsia, preterm birth, low birth weight, and small-for-gestational-age infants, as specified in Sections 2.2

Section and Topic	Item #	Checklist item	Location where item is reported
			and 2.4.
	10b	List and define all other variables for which data were sought (e.g. participant and intervention characteristics, funding sources). Describe any assumptions made about any missing or unclear information.	Extracted variables included study characteristics, population demographics, genetic polymorphisms, exposure sources, and methodological features, as reported in Section 2.4.
Study risk of bias assessment	11	Specify the methods used to assess risk of bias in the included studies, including details of the tool(s) used, how many reviewers assessed each study and whether they worked independently, and if applicable, details of automation tools used in the process.	Methodological quality and risk of bias were assessed using the Newcastle–Ottawa Scale (NOS) by independent reviewers, as described in Section 2.4.
Effect measures	12	Specify for each outcome the effect measure(s) (e.g. risk ratio, mean difference) used in the synthesis or presentation of results.	Effect measures included mean differences (MDs) with 95% confidence intervals for continuous outcomes, as described in Section 2.5.
Synthesis methods	13a	Describe the processes used to decide which studies were eligible for each synthesis (e.g. tabulating the study intervention characteristics and comparing against the planned groups for each synthesis (item #5)).	Studies were included in each synthesis based on genetic comparability, exposure assessment methods, and outcome definitions, as described in Sections 2.2 and 2.5.
	13b	Describe any methods required to prepare the data for presentation or synthesis, such as handling of missing summary statistics, or data conversions.	Data transformations, including conversion of geometric means to mean differences and standard deviations, were performed as described in Section 2.4.
	13c	Describe any methods used to tabulate or visually display results of individual studies and syntheses.	Results were presented using structured tables, forest plots, and PRISMA flow diagrams, as reported in the Results section.
	13d	Describe any methods used to synthesize results and provide a rationale for the choice(s). If meta-analysis was performed, describe the model(s), method(s) to identify the presence and extent of statistical heterogeneity, and software package(s) used.	Meta-analyses were conducted using RevMan

Section and Topic	Item #	Checklist item	Location where item is reported
			5.4 with fixed- or random-effects models based on heterogeneity assessed by I² and Cochran's Q test, as described in Section 2.5.
	13e	Describe any methods used to explore possible causes of heterogeneity among study results (e.g. subgroup analysis, meta-regression).	NA/ Statistical heterogeneity was assessed using I² statistics, as reported in Section 2.5.
	13f	Describe any sensitivity analyses conducted to assess robustness of the synthesized results.	Sensitivity analyses were conducted by excluding studies with high risk of bias (NOS < 7), in Section 2.4.
Reporting bias assessment	14	Describe any methods used to assess risk of bias due to missing results in a synthesis (arising from reporting biases).	Publication bias was assessed using funnel plots and Egger's test, in Section 2.5.
Certainty assessment	15	Describe any methods used to assess certainty (or confidence) in the body of evidence for an outcome.	NA
**RESULTS**			
Study selection	16a	Describe the results of the search and selection process, from the number of records identified in the search to the number of studies included in the review, ideally using a flow diagram.	Results of the study identification and selection process are presented using a PRISMA flow diagram (Figure 1), including numbers of records identified, screened, excluded, and included.
	16b	Cite studies that might appear to meet the inclusion criteria, but which were excluded, and explain why they were excluded.	Studies excluded after full-text assessment and reasons for exclusion are described in Section 3.1 and illustrated in the PRISMA flow diagram.
Study characteristics	17	Cite each included study and present its characteristics.	Table 1
Risk of bias in studies	18	Present assessments of risk of bias for each included study.	Newcastle–Ottawa Scale (NOS), in Supplementary Table S1.

Section and Topic	Item #	Checklist item	Location where item is reported
Results of individual studies	19	For all outcomes, present, for each study: (a) summary statistics for each group (where appropriate) and (b) an effect estimate and its precision (e.g. confidence/credible interval), ideally using structured tables or plots.	Tables 2 and 3.
Results of syntheses	20a	For each synthesis, briefly summarise the characteristics and risk of bias among contributing studies.	Characteristics of studies included in each synthesis and their methodological quality are summarized in Tables 1–3 and Supplementary Table S1.
	20b	Present results of all statistical syntheses conducted. If meta-analysis was done, present for each the summary estimate and its precision (e.g. confidence/credible interval) and measures of statistical heterogeneity. If comparing groups, describe the direction of the effect.	Section 3.3 and Figure 2.
	20c	Present results of all investigations of possible causes of heterogeneity among study results.	Heterogeneity was assessed using I^2 statistics; further subgroup or meta-regression analyses were NA due to the limited number of comparable studies.
	20d	Present results of all sensitivity analyses conducted to assess the robustness of the synthesized results.	Not clearly
Reporting biases	21	Present assessments of risk of bias due to missing results (arising from reporting biases) for each synthesis assessed.	Publication bias was assessed using funnel plots and Egger's test when applicable; detailed quantitative assessment was NA due to the small number of studies.
Certainty of evidence	22	Present assessments of certainty (or confidence) in the body of evidence for each outcome assessed.	NA
**DISCUSSION**			
Discussion	23a	Provide a general interpretation of the results in the context of other evidence.	General interpretation of findings in relation to existing evidence is discussed in Discussion paragraphs 1–6, comparing current results with previous epidemiological and mechanistic studies on Hg toxicity, genetic polymorphisms, and perinatal outcomes.

Section and Topic	Item #	Checklist item	Location where item is reported
	23b	Discuss any limitations of the evidence included in the review.	Limitations of the included evidence, including heterogeneity of biomarkers, study designs, sample sizes, and confounding factors, are addressed in Discussion paragraph 13
	23c	Discuss any limitations of the review processes used.	Discussion paragraph 13
	23d	Discuss implications of the results for practice, policy, and future research.	Well described, discussion paragraphs 10–14
**OTHER INFORMATION**			
Registration and protocol	24a	Provide registration information for the review, including register name and registration number, or state that the review was not registered.	PROSPERO (CRD420251016754)
	24b	Indicate where the review protocol can be accessed, or state that a protocol was not prepared.	Reported in Methods, paragraph 1.
	24c	Describe and explain any amendments to information provided at registration or in the protocol.	NA
Support	25	Describe sources of financial or non-financial support for the review, and the role of the funders or sponsors in the review.	This research was funded by the Indonesian Collaborative Research Grant, Institute for Research and Community Service, No. 325-22/UN7.D2/PP/V/2025.
Competing interests	26	Declare any competing interests of review authors.	Page 13
Availability of data, code and other materials	27	Report which of the following are publicly available and where they can be found: template data collection forms; data extracted from included studies; data used for all analyses; analytic code; any other materials used in the review.	Page 13

From: Page MJ, McKenzie JE, Bossuyt PM, Boutron I, Hoffmann TC, Mulrow CD, et al. The PRISMA 2020 statement: an updated guideline for reporting systematic reviews. BMJ 2021;372:n71. doi: 10.1136/bmj.n71. This work is licensed under CC BY 4.0. To view a copy of this license, visit https://creativecommons.org/licenses/by/4.0/
